# Left inguinal dedifferentiated liposarcoma and primary unclassified sarcoma of the left lung as synchronous multiple sarcomas: a case report

**DOI:** 10.1186/s40792-024-02043-0

**Published:** 2024-10-28

**Authors:** Masao Kobayashi, Hidetoshi Satomi, Hisaya Chikaraishi, Hironobu Samejima, Julian Horiguchi, Ryu Kanzaki, Tomohiro Maniwa, Keiichiro Honma, Jiro Okami

**Affiliations:** 1 Department of General Thoracic Surgery, Osaka International Cancer Institute, 3-1-69, Otemae, Chuo-Ku, Osaka, Osaka 540-0008 Japan; 2 Department of Pathology, Osaka International Cancer Institute, 3-1-69, Otemae, Chuo-Ku, Osaka, Osaka 540-0008 Japan

**Keywords:** Dedifferentiated liposarcoma, Primary pulmonary sarcoma, Pulmonary metastasis, Synchronous multiple sarcomas, Soft tissue sarcoma

## Abstract

**Background:**

Pulmonary nodules in patients with soft tissue sarcomas are likely pulmonary metastases, whereas synchronous primary pulmonary sarcomas are rare. Without surgery, determining whether a solitary pulmonary nodule is a primary or metastatic nodule is difficult. Herein, we report a rare case of a primary pulmonary sarcoma that presented synchronously with a primary dedifferentiated liposarcoma.

**Case presentation:**

A 77-year-old man presented to another hospital with left inguinal swelling and a suspected recurrent inguinal hernia. Computed tomography revealed a left inguinal mass and pure-solid nodule in the left lung and the patient was referred to our hospital for detailed examination and treatment. The inguinal mass was pathologically diagnosed as a dedifferentiated liposarcoma using needle biopsy, whereas bronchoscopic biopsy revealed histological findings suggestive of a sarcoma; however, the primary site could not be determined. Positron emission tomography–computed tomography revealed no high-accumulation lesions except for the two sarcomas. We decided to perform surgery on both sarcomas for diagnostic and curative purposes. The surgical specimens showed that the two sarcomas were different. Based on the immunohistochemical staining findings of MDM2, a left inguinal dedifferentiated liposarcoma and primary pulmonary unclassified sarcoma were diagnosed. The patient displayed no evidence of recurrence 1 year after surgery.

**Conclusions:**

We encountered a rare case of synchronous multiple primary sarcomas, one presenting in the lung and the other in the soft tissue. Surgery was required to achieve a definitive diagnosis for the patient, who achieved disease-free survival at 1 year. This case suggests that proactive resection of pulmonary nodules in patients with soft tissue sarcomas may be feasible as a diagnostic treatment if complete resection is achieved.

## Background

Primary pulmonary sarcoma (PPS) is a rare tumor type, accounting for 0.013–1.1% of all malignant lung tumors [1]. Spraker et al. reported that PPSs have a poor prognosis [5-year overall survival (OS), 35%] [2]. In contrast, liposarcoma is the most common soft tissue sarcoma, accounting for approximately 20% of all mesenchymal malignancies [3]. Dedifferentiated liposarcoma (DDLPS) is a high-grade sarcoma with a poor prognosis (5-year OS rate, 51.5%) [4]. The most common distant metastatic site for DDLPS is the lung [5].

Herein, we report a rare case of synchronous multiple sarcomas, which may be confused with the more commonly observed primary sarcoma and its coexisting pulmonary metastasis. To the best of our knowledge, this is the first report of synchronous lung and soft tissue sarcomas.

## Case presentation

**Fig. 1:**
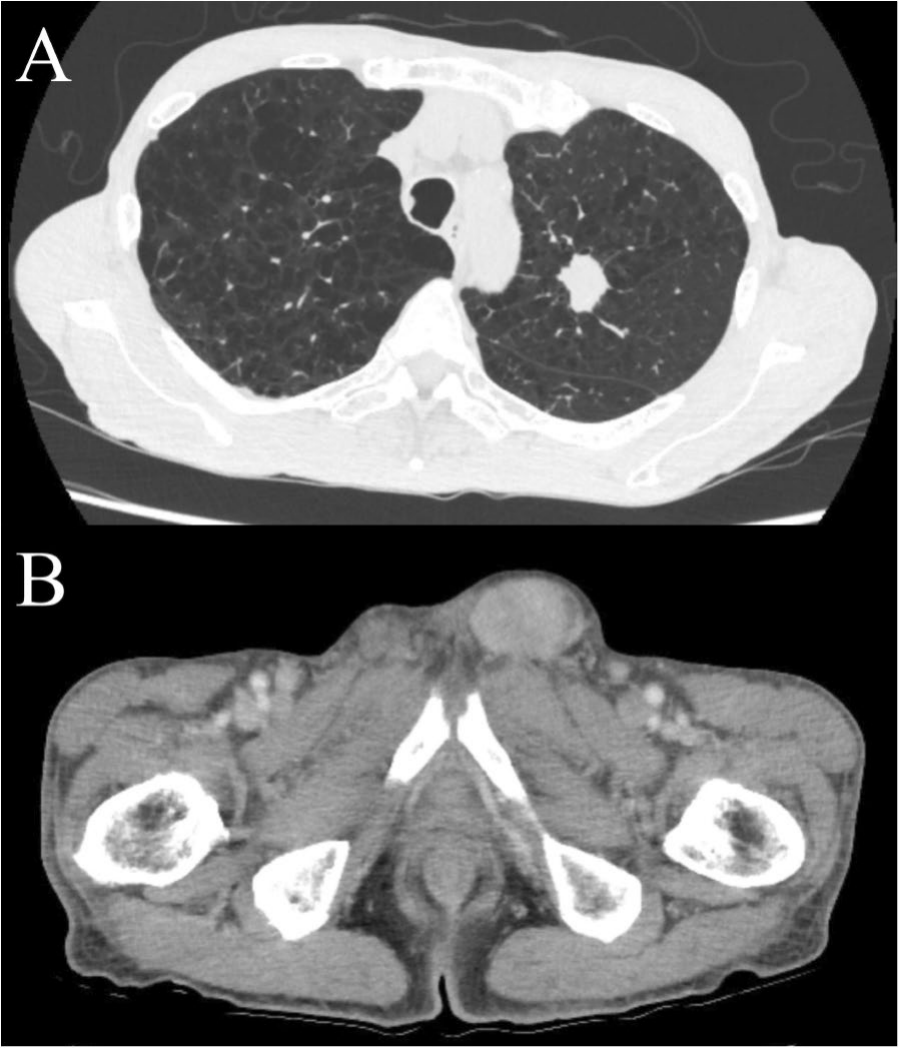
Computed tomography findings of the pulmonary nodule and inguinal solid mass. A computed tomography scan reveals a pure-solid pulmonary nodule (23 × 20 × 25 mm) in the left upper lobe (**A**) and solid mass (50 × 30 × 55 mm) in the left inguinal area (**B**)

**Fig. 2:**
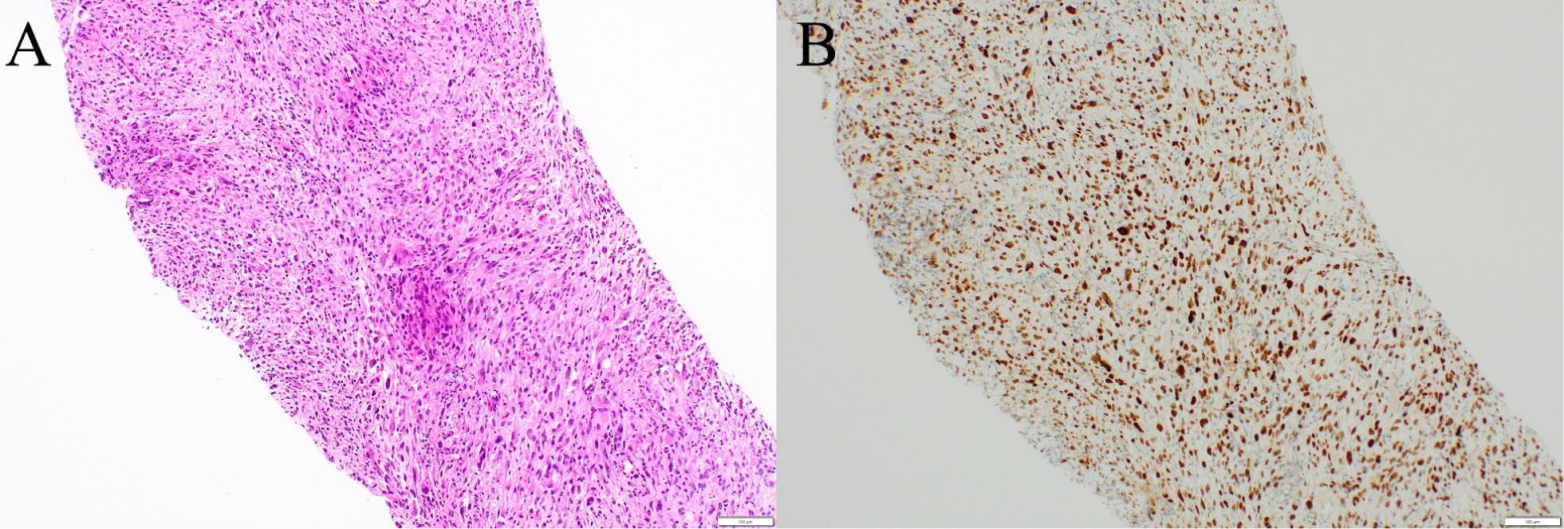
Pathological examination of needle biopsy of the inguinal mass showed stromal cell proliferation (**A**) and positive staining for MDM2 (**B**); hence, the tumor was diagnosed as a DDLPS

**Fig. 3:**
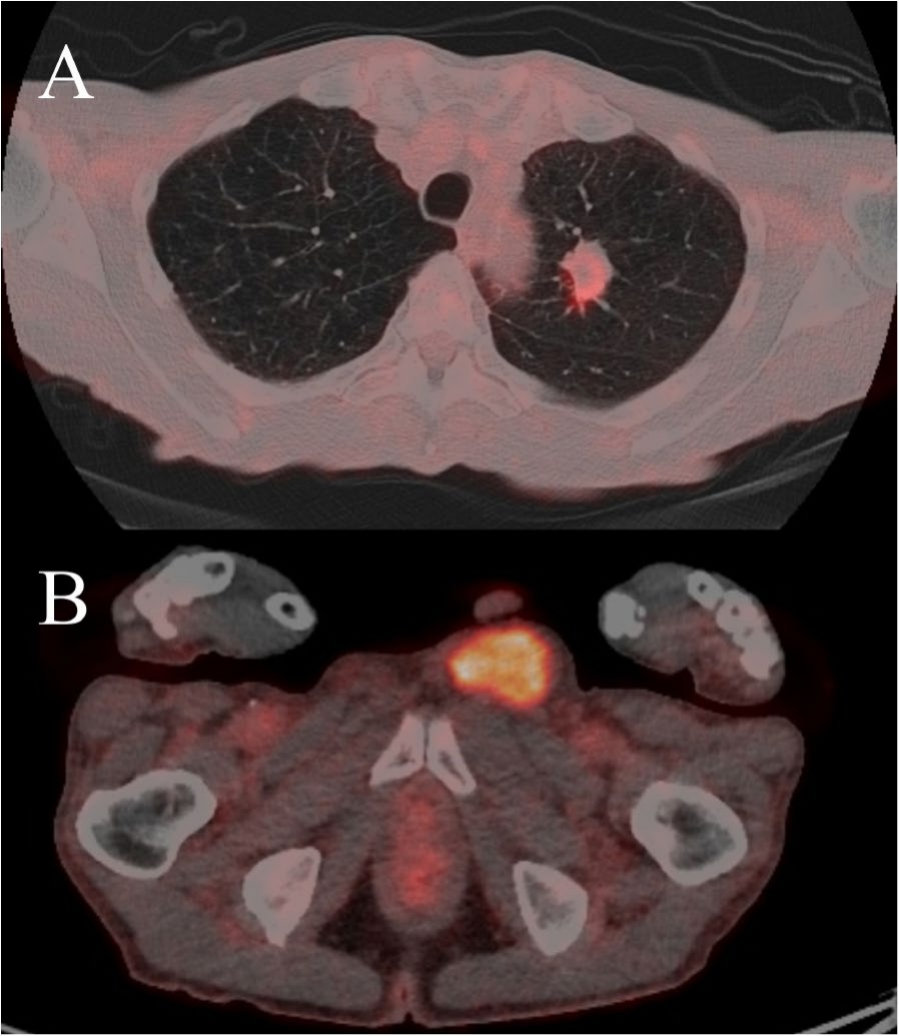
Positron emission tomography–computed tomography findings of the two sarcomas. A scan shows a high accumulation of both the pulmonary sarcoma (SUVmax, 3.5) (**A**), and inguinal dedifferentiated liposarcoma (SUVmax, 7.7) (**B**), with no other abnormal accumulation. SUVmax, maximum standard uptake value

**Fig. 4:**
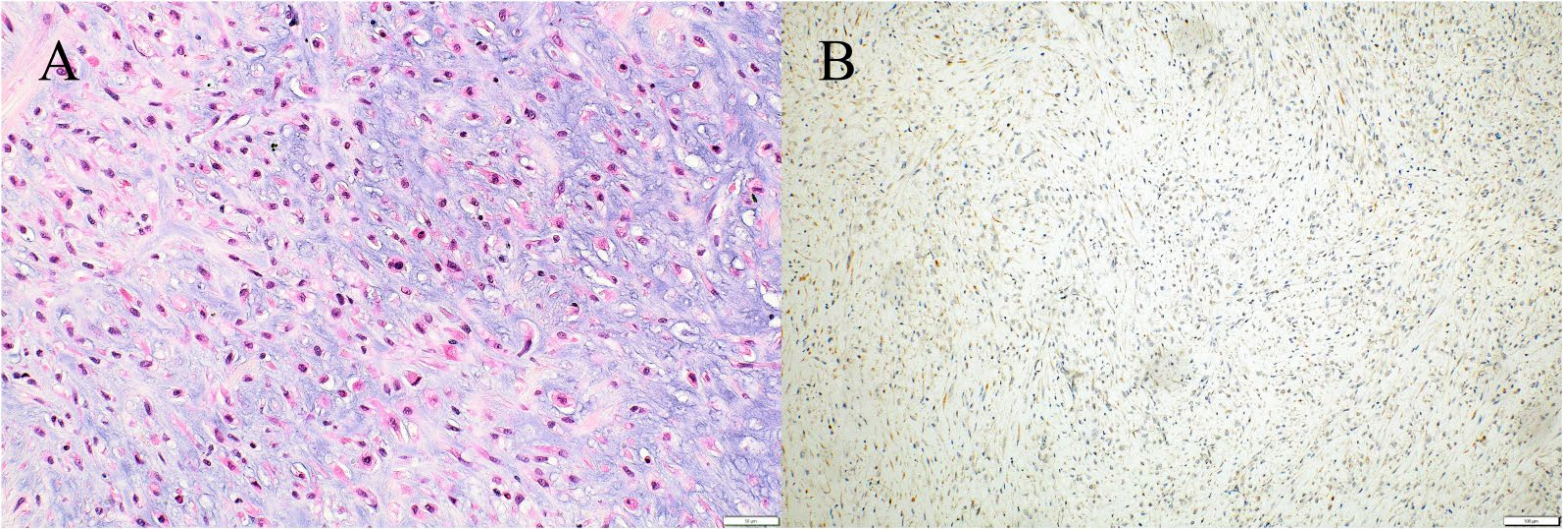
Histopathological examination of the pulmonary sarcoma revealed stromal cell proliferation with differentiation into the cartilage (**A**) and negative staining for MDM2 (**B**)

**Fig. 5:**
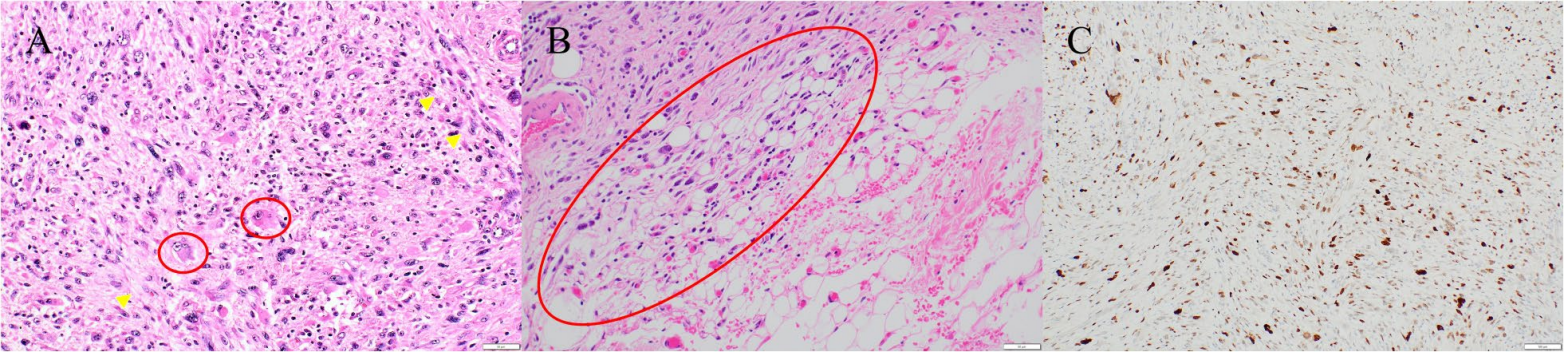
Inguinal DDLPS showed stromal cell (yellow arrow) combined with round cell proliferation (red circle) (**A**), along with well differentiated liposarcoma (red circle) (**B**). The immunohistochemistry findings showed positive staining for MDM2 (**C**)

**Fig. 6:**
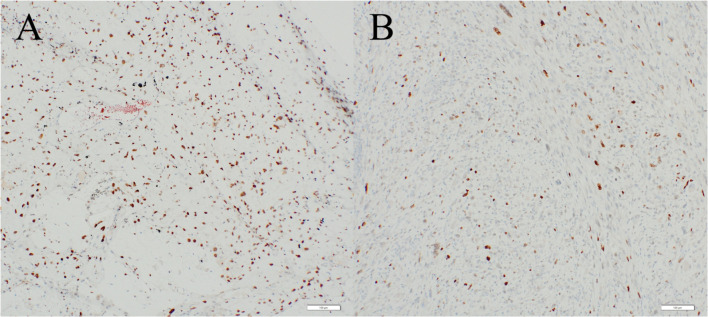
Immunohistochemical examination showed p53 overexpression in the primary pulmonary unclassified sarcoma (**A**), while only a few cells expressed positivity for p53 (**B**)

A 77-year-old man presented to another hospital with left inguinal swelling and a suspected recurrent inguinal hernia. The patient’s medical history was significant for hernioplasty for a left inguinal hernia, pancreatoduodenectomy for an intraductal papillary mucinous neoplasm, and diabetes mellitus. The patient had a smoking history of 46 packs pack-year, and he had no family history of malignancies. Computed tomography images revealed a left inguinal mass and pulmonary nodule in the left lung, and the patient was referred to our hospital for detailed examination and treatment. Based on the computed tomography findings, a pure-solid pulmonary nodule (23 × 20 × 25 mm) was found in the left upper lobe and a solid mass (50 × 30 × 55 mm) was found in the left inguinal area (Fig. 1). Laboratory findings revealed no abnormalities in serum tumor markers (including squamous cell carcinoma antigen, 0.6 ng/mL; cytokeratin fragment 19, 2.0 ng/mL; and pro-gastrin-releasing peptide, 29.2 pg/mL) except for a slight elevation of carcinoembryonic antigen (5.1 ng/mL). Needle biopsy of the inguinal mass revealed stromal cell proliferation (Fig. 2A) and positive staining for MDM2 (Fig. 2B). Hence, the tumor was diagnosed as a DDLPS. Conversely, bronchoscopic biopsy failed to determine the histological subtype of the sarcoma or the primary site (i.e., lung or soft tissue) owing to insufficient specimens, and immunohistochemical examination was not performed. Positron emission tomography–computed tomography (PET–CT) indicated a high accumulation of both pulmonary sarcoma (maximum standardized uptake value [SUVmax], 3.5) and inguinal DDLPS (SUVmax, 7.7), with no other abnormal accumulation (Fig. 3). After consulting the cancer board at our hospital, surgical resection of both sarcomas was planned for definitive diagnosis and complete removal. Video-assisted left upper segmentectomy, wide excision of the left inguinal soft tissue, and inguinal orchiectomy were performed. Histopathological examination of the pulmonary sarcoma revealed stromal cell proliferation with differentiation into the cartilage (Fig. 4A), and negative staining for MDM2 (Fig. 4B) as well as negative staining for smooth muscle actin, desmin, CD 34, S100, SS18-SSX and STAT6 (data not shown). Based on the lack of distinguishing immunohistochemical features for any well-defined histological type, the tumor was diagnosed as primary pulmonary unclassified sarcoma. The total tumor size was 22 mm without pleural, venous, or lymphatic invasion or lymph node metastasis, and the pathological stage was classified as IA3 (T1cN0M0, TNM staging 8th edition). In contrast, the inguinal DDLPS showed stromal cells combined with round cell proliferation (Fig. 5A), along with well differentiated liposarcoma (Fig. 5B), the same findings seen on the needle biopsy specimen. The immunohistochemistry findings showed positive staining for MDM2 (Fig. 5C), smooth muscle actin, and desmin (data not shown). The immunohistochemical examination also showed p53 overexpression in the primary pulmonary unclassified sarcoma (Fig. 6A), while only a few cells expressed positivity for p53 (Fig. 6B). Therefore, the pulmonary and inguinal sarcomas were concluded to be synchronous multiple sarcomas. The patient was followed-up every 6 months using computed tomography and had experienced no recurrence 1 year after surgery.

## Discussion

Cases of multiple sarcomas in a single patient are extremely rare. A report by Lex et al. revealed that only 6 (0.08%) of 7351 patients with sarcomas experienced synchronous or metachronous multiple sarcomas [6]. Another report by Grobmyer described that the incidence was only 0.2% (9 of 5505 patients) [7]. Genetic disposition has been reported to be a possible risk factor for multiple primary malignancies in a single patient [8]. In the present case, we retrospectively reviewed the specimens and identified p53 protein overexpression in the pulmonary sarcoma, indicating a p53 gene mutation. In contrast, DDLPS revealed only a few tumor cells that were positive for p53. As a result, the two sarcomas were not considered to share the same genetic mutation. Moreover, patient did not have a family history of malignancy, and was therefore less likely to have predisposing cancerous diseases such as Li-Fraumeni syndrome.

With the exception of mulcentric sarcomas, reports about patients with synchronous multiple sarcomas are limited. A summary of these reports is presented in Table 1 [7, 9–17]. In general, most soft tissue sarcomas have a tendency toward lung metastasis [18], and multiple pulmonary nodules are more likely to be pulmonary metastases. However, regarding solitary pulmonary nodules, it may be worth distinguishing them from synchronous malignancies as in the present case.

**Table 1:** Reported cases of synchronous multiple sarcomas except multicentric sarcomas

Case	Author	Year	Age	Sex	First sarcoma	Site	Treatment for the first sarcoma	Second sarcoma	Site	Perioperative treatment for the second sarcoma	Outcome	Overall survival from diagnosis of the fist sarcoma
1	Grobmyer et al. [7]	2004	77	Male	DDLPS	Extremity	Surgery	Gastrointestinal stromal tumor	N/A	Surgery	Alive without disease	1 year
2	Grobmyer et al. [7]	2004	51	Female	Well differentiated liposarcoma	Extremity	Surgery	Leiomyosarcoma	Retroperitoneal	Surgery	Alive without disease	2 months
3	Sato et al. [9]	2004	72	Male	Liposarcoma	Peritoneal	Surgery	Liposarcoma	Stomach and multiple lesions	Surgery	Alive without disease	12 months
4	Cui et al.[10]	2008	40	Female	Granulocytic sarcoma	Brest	Surgery	Granulocytic sarcoma	Spine	Surgery followed by adjuvant chemotherapy	Alive without disease	20 months
5	Gandhi et al. [11]	2012	61	Male	Rhabdomyosarcomas	Esophagus	Chemoradiotherapy followed by surgery and postoperative chemotherapy	Rhabdomyosarcomas	Stomach	Chemoradiotherapy followed by surgery and postoperative chemotherapy	Alive without disease	9 months
6	Brabuscia et al. [12]	2013	82	Female	Myofibroblastoma	Brest	Surgery	Osteosarcoma	Breast	Surgery	Alive without disease	4 years
7	Kutluk et al. [13]	2019	25	Male	Chondrosarcoma	6th rib	Surgery	Chondrosarcoma	2nd rib	Surgery	Alive without disease	43 months
8	Nie et al. [14]	2021	71	Male	DDLPS	Kidney	Surgery	Well differentiated liposarcoma	Retroperitoneal	Surgery	N/A	N/A
9	Diamantis et al. [15]	2021	86	Male	Well differentiated liposarcoma	Stomach	Surgery	Gastrointestinal stromal tumor	Stomach	Surgery	Alive without disease	5 years
10	Diamantis et al. [15]	2021	66	Male	Well differentiated liposarcoma	Retroperitoneal	Surgery followed by adjuvant chemotherapy	Gastrointestinal stromal tumor	Stomach	Surgery	Died of other diseases	13 months
11	Mikhael et al. [16]	2022	80	Female	Undifferentiated pleomorphic sarcoma	Thigh	Surgery followed by radiation therapy	Liposarcoma	Retroperitoneal	Surgery	N/A	N/A
12	Kapagan et al. [17]	2024	59	Male	Angiosarcoma	Stomach	Chemotherapy	Angiosarcoma	Rectum	Chemotherapy	Died of disease	4 months

*N/A* Not available

Porte et al. reported that only 4 (22.2%) of 18 patients with PPS were correctly diagnosed using bronchoscopic biopsy specimens [19], suggesting the difficulty of preoperative diagnosis by bronchoscopy. In addition, although imaging modalities such as CT or PET–CT reveal the anatomical characteristics or the biological features, these features are non-specific and are observed in any type of lung malignancy [19, 20]. Parkes et al. reported that an SUVmax of 4 was identified as the cutoff point in PET–CT findings to distinguish DDLPS from well-differentiated liposarcoma [21], however, a case report by Imamura et al. described pulmonary metastasis of DDLPS without 18F-fluorodeoxyglucose uptake [22]. Therefore, it may be difficult to make a definitive diagnosis without surgical resection in some PPS cases such as the present case. Although bronchoscopic re-biopsy was an option, more specimens were not expected to be obtained using the same modality. Hence, surgery for diagnostic and curative purposes was considered acceptable in the present case.

The mainstay of the management of DDLPS is surgical resection with safety margins [24]. In the present case, due to the location of inguinal DDLPS adjacent to the inguinal canal, combined resection of the left spermatic cord and testicle was performed. Moreover, complete resection of PPS is the optimal curative approach [1, 19, 24]. In the present case, the patient was old and had a heavy smoking history, a history of diabetes mellitus, and a past surgical history of pancreatoduodenectomy. Thus, he was considered high risk for lobectomy. Considering the anatomical location of the tumor, sufficient surgical margins could be secured, and segmentectomy was performed. In fact, a large-scale report of 695 patients undergoing surgery for PPS described that the type of resection (lobar or sublobar) was not predictive of survival [25]. Therefore, sublobar resection may be feasible in specific cases.

According to Spraker et al., PPSs (5-year OS, 35%) have a poorer prognosis than sarcomas of the extremities (5-year OS, 71%) [2]. Complete resection of PPSs has been reported to prolong survival [24]. Although a DDLPS with distant metastases has an even poorer prognosis (5-year OS, 12.1%) [26], patients undergoing resection of the metastatic DDLPS lesions had better prognosis than those with unresectable disease [27]. Hence, in the present case, resection of the sarcomas of both the lungs and groin was feasible for curative indications. The resected specimens provided a definitive diagnosis of synchronous multiple sarcomas. The present case indicates that proactive resection of pulmonary nodules in patients with soft tissue sarcomas may be feasible as a diagnostic treatment if complete resection is achieved.

Although no recurrence was observed in the present case, both sarcomas have the potential for recurrence. In case of appearance of recurrent lesions, it is essential to distinguish the primary sarcoma (DDLPS or PPS). Metastasectomy for DDLPS is feasible because DDLPS has been considered as insensitive to chemotherapy [3, 5]. Cytotoxic chemotherapy regimens are available for patients with DDLPS with unresectable recurrence, but the efficacy is limited [4]. Conversely, the therapeutic strategy for recurrent PPS remains to be elucidated, and cytotoxic chemotherapy regimens are options [1]. In the present case, chemotherapy options should be carefully selected based on the general condition of the patient.

## Conclusion

In patients with soft tissue sarcomas who develop pulmonary nodules, these nodules are typically found to be pulmonary metastases. Conversely, synchronous multiple sarcomas are rare. A solitary pulmonary nodule in patients with soft tissue sarcomas may lead to confusion regarding diagnosis; however, complete resection of the pulmonary nodule may be feasible to achieve a diagnostic treatment.

## Data Availability

The authors declare that all data within this article are available upon reasonable request. The data supporting the findings of this study are available from the corresponding author upon reasonable request.

## Abbreviations

PPS: Primary pulmonary sarcoma
OS: Overall survival
DDLPS: Dedifferentiated liposarcoma
PET–CT: Positron emission tomography–computed tomography
SUVmax: Maximum standard uptake value
